# The Pathognomonic Signs of Perforating Appendicitis

**Published:** 1892-08

**Authors:** 


THE PATHOGNOMONIC SIGNS OF PERFORATING APPENDICITIS.—Dr. Simon Baruch (*Med. Record*) emphasizes the point that symptoms of shock, carefully looked for, may always be found in perforating appendicitis. These are as follows: The countenance is anxious, the finger-tips, nose and ears are cool; pulse and respiration are out of proportion to temperature, the right inguinal region is very tender, the patient usually lies with the right leg drawn up. Guided by them, Dr. Baruch opposed the views of an experienced physician in one case, insisting upon the operation; and in another did not approve of the operation advised by an experienced surgeon. In both cases his reliance on these pathognomonic signs proved useful to the patient. On the ground of his own experience, as well as that of others, the author urges that when perforating appendicitis is diagnosed, either positively or probably, an immediate operation to remove the exciting cause is as imperative as ligation of the vessel in hemorrhage.

The fact that laparotomies are now constantly performed, under strict asepsis, with absolute safety, should induce the attendant to clear up a doubtful diagnosis of perforating appendicitis by an operation before septic peritonitis forbids it.—*Int. Jour. of Surgery.*

